# Fabrication of Ropivacaine/Dexamethasone-Eluting Poly(D, L-lactide-co-glycolide) Microparticles via Electrospraying Technique for Postoperational Pain Control

**DOI:** 10.3390/polym14040702

**Published:** 2022-02-11

**Authors:** Shih-Jyun Shen, Ying-Chao Chou, Shih-Chieh Hsu, Yu-Ting Lin, Chia-Jung Lu, Shih-Jung Liu

**Affiliations:** 1 Department of Mechanical Engineering, Chang Gung University, Taoyuan 33302, Taiwan; m8420@cgmh.org.tw (S.-J.S.); runner484@gmail.com (S.-C.H.); yutinna9876@mail.cgu.edu.tw (Y.-T.L.); happy2231017@mail.cgu.edu.tw (C.-J.L.); 2 Department of Anesthesiology, Chang Gung Memorial Hospital at Linkou, Taoyuan 33305, Taiwan; 3 Department of Orthopedic Surgery, Bone and Joint Research Center, Chang Gung Memorial Hospital at Linkou, Taoyuan 33305, Taiwan; enjoycu@ms22.hinet.net

**Keywords:** electrospraying, microparticles, ropivacaine, dexamethasone, sustained release

## Abstract

Microencapsulation plays an important role in biomedical technology owing to its particular and attractive characteristics. In this work, we developed ropivacaine and dexamethasone loaded poly(D, L-lactide-co-glycolide) (PLGA) microparticles via electrospraying technique and investigated the release behavior of electrosprayed microparticles. The particle morphology of sprayed particles was assessed using scanning electron microscopy (SEM). The in vitro drug release kinetics were evaluated employing an elution method, and the in vivo pharmaceutical release as well as its efficacy on pain relief were tested using an animal activity model. The microscopic observation suggested that sprayed microparticles exhibit a size distribution of 5–6 µm. Fourier-transform infrared spectrometry and differential scanning calorimetry demonstrated the successful incorporation of pharmaceuticals in the PLGA particulates. The drugs-loaded particles discharged sustainably high concentrations of ropivacaine and dexamethasone at the target region in vivo for over two weeks, and the drug levels in the blood remained low. By adopting the electrospraying technique, we were able to prepare drug-embedded polymeric microparticles with effectiveness and with a sustainable capability for postoperative pain control.

## 1. Introduction

Despite the advances in pharmaceutical technologies, the delivery of sustained drug concentrations to the target site remains as a challenge. Polymeric microencapsulation plays an important role in biomedical technology owing to its particular and attractive characteristics, involving compatibility, durability, target specificity, consistent encapsulation, superior compliance, and controllable sustained drug releases capable of minimizing the toxicity and dosage frequency [1]. Microparticles generally range between 1 and 1000 µm and act as versatile drug delivery systems with well-defined physiological and pharmacokinetic properties to promote effectiveness, tolerability, and patient compliance. One major advantage of microparticles over nanocarriers lies in that they do not pass through the interstitium over the dimension of 100 nm conveyed by the lymph and only perform their functions locally [2].

**Figure 1:**
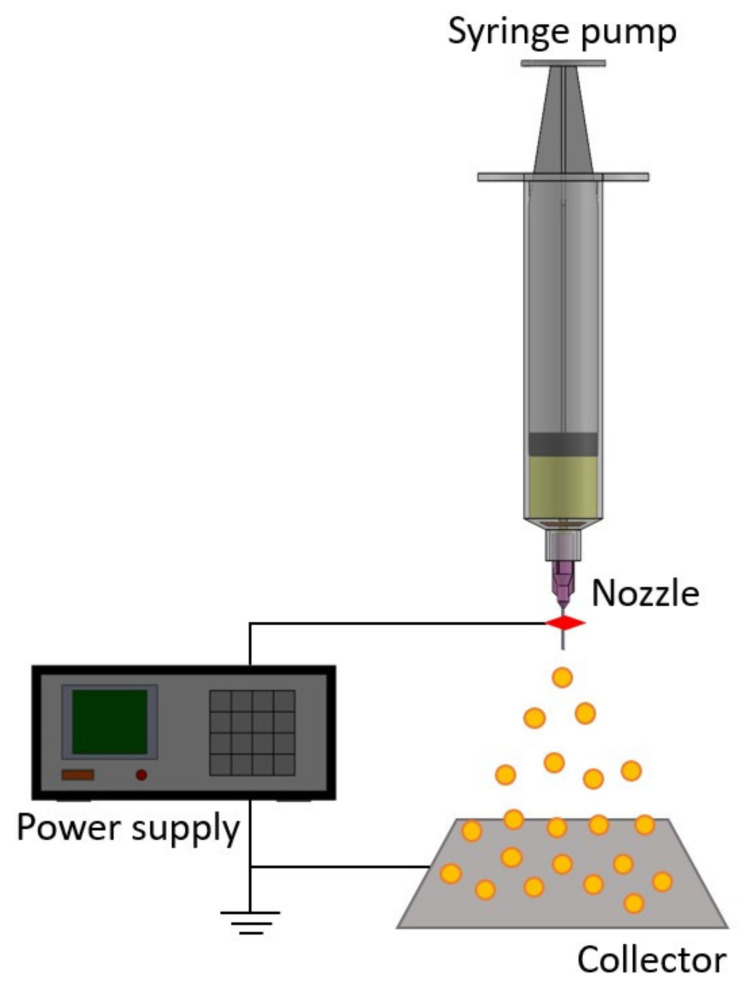
The schematic of the electrospraying setup.

Electrospraying is a versatile technique capable of producing micro- and nano-polymeric particles for drug delivery [3,4]. In the procedure, the electrically charged solution is ejected out of a nozzle, overcoming the surface tension of the polymeric solution, and split into tiny droplets (Figure 1). These charged droplets counteract with one another, in turn preventing the consolidation of droplets and leading to the formation of tiny particles with a uniform size distribution [5]. Compared with the other traditional schemes for preparing polymeric microparticles, the coulombic repulsion among the highly charged sprayed droplets leads to self-dispersion particles without coalescence. In addition, owing to the absence of exterior media that permit the dissolution or migration of incorporated drugs, the encapsulation efficiency by electrospraying can be optimized [6]. The preparation of core/shell-structured microspheres is also feasible using this novel technology [7].

Post-surgery pain remains undertreated in most regions globally, particularly in developing countries, affecting 47–100% of patients [8]. The management of postoperative pain is not easy because of complex etiologies and patients’ subjective experiences. It is important to identify the type of operation before managing the pain related to surgery. The American Pain Society suggested multimodal analgesia, a variety of analgesic medications and techniques for the treatment of postoperative pain in children or adults [9]. Abdominal surgeries are operations resulting in a high degree of pain [10]. Regional analgesia techniques, such as epidural analgesia or peripheral nerve block, are recommended in most enhanced recovery after surgery protocols to reduce opioid consumption [9,11]. Many types of peripheral nerve block have been effective and widely used in patients receiving abdominal surgery, such as transversus abdominal plane block, rectus sheath block, quadratus lumborum block, etc. However, most of the drugs used in previous techniques do not act adequately long.

Degradable poly[(D, L)-lactide-co-glycolide] (PLGA) incorporated with lidocaine was proved to offer extended analgesia [12,13]. Other research also revealed that lidocaine and bupivacaine play an important role in multimodal analgesia for laparotomy wounds in the mouse grimace scale and von Frey test [14]. However, the duration of the analgesic effect was limited by the choice of drugs.

This study developed ropivacaine and dexamethasone-incorporated PLGA microparticles via electrospraying technique. Ropivacaine is a local anesthetic belonging to the amino amide group with less cardiovascular and central nerve toxicity than bupivacaine [15]. Dexamethasone is a glucocorticoid medication with an anti-inflammatory effect and with local anesthetics analgesia-prolonging effects [16,17] that reduces the possibility of chronic pain [18]. After spraying, the morphological structure of drug-loaded PLGA particulates was characterized by scanning electron microscopy. In vitro discharge behavior was assessed by an elution method and by a high-performance liquid chromatography (HPLC) analysis. Additionally, the microspheres were injected into the muscular layer near the laparotomy wound. The in vivo drug concentration and the postoperative activity of rats were analyzed. The histology examination of the tissue was also accomplished.

## 2. Materials and Methods

### 2.1. Production of Analgesics-Eluting Microparticles

PLGAs (50:50), ropivacaine, dexamethasone, and dichloromethane (DCM) were provided by Sigma-Aldrich (St. Louis, MO, USA).

Drugs-loaded microcarriers, with two distinct polymer-to-drug ratios, were prepared utilizing a lab-scale electrospraying apparatus, composed of a syringe with a needle (inner diameter = 60 m), a ground collection tray, and a high-voltage power supply. For the manufacture of microparticles with a polymer:drug 3:1 ratio, PLGA (600 mg) and drugs (150 mg ropivacaine and 42 mg dexamethasone) were agitatedly blended with 10 mL of DCM for 60 mins. The mixture was delivered by a syringe pump with a flow speed of 1.0 mL/h. The syringe needle was attached to the power supply with a DC voltage of 8 kV. The interspace between the needle tip and the collection tray was 12 cm. The production of microparticles with a polymer:drug 4:1 ratio follows the same preparatory procedure, except that PLGA (300 mg) and drugs (75 mg ropivacaine and 21 mg dexamethasone) were dissolved in 5 mL of DCM.

### 2.2. Scanning Electron Microscopy (SEM)

Drug-loaded microparticles were first sputtered with gold and then placed under an SEM (JSM-7500F, Jeol, Tokyo, Japan) for observation. The dimension distribution of the sprayed microparticles was acquired by analyzing 50 randomly chosen particles for each image (*n* = 5).

### 2.3. Fourier-Transform Infrared Spectrometry (FTIR)

FTIR analysis was accomplished on a spectrometer (Nicolet iS5, Thermo Fisher, Waltham, MA, USA) with a resolution of 4 cm^−1^ (32 scans). Drugs-loaded microparticles were incorporated in compressed KBr disks, and the assay was evaluated from 500 to 4000 cm^−1^.

### 2.4. Differential Scanning Calorimetry

The thermal properties of virgin PLGA and ropivacaine/dexamethasone loaded PLGA particles were estimated by a TA-DSC25 differential scanning calorimeter (TA Instruments, New Castle, DE, USA). The scanning temperature ranged from 30 to 350 °C and the specimens were heated at 10 °C/min.

### 2.5. Discharge of Pharmaceuticals In Vitro

The discharge of pharmaceuticals from PLGA particles in vitro was assessed using an elution scheme [19,20] and an HPLC assay on an L-2200 System (Hitachi, Tokyo, Japan) along with a Discovery BIO wide Pore C18 25 cm × 4.6 mm × 5 µm column. Phosphate-buffered saline (PBS, pH 7.4) was used as the dissolution medium. The microparticles were placed in glass test tubes (*n* = 3) with 1 mL of PBS. All tubes were incubated at 37 °C. The dissolution medium was collected and analyzed every 24 h. Fresh PBS (1 mL) was then added for the next 24 h. The drug concentrations in the buffer for the elution studies were determined using HPLC. The process was repeated for 14 days.

The mobile phase used for ropivacaine contained acetonitrile:phosphate buffer (60:40, *v:v*). The absorbency was monitored at a wavelength of 240 nm, and the flow rate was 1.2 mL/min. The assay was conducted at 30 °C. The mobile phase used for dexamethasone included methanol:phosphate buffer (60/40, *v*/*v*). The absorbency was evaluated at 25 °C with a wavelength of 270 nm, and the flow speed was maintained at 1.5 mL/min.

### 2.6. Rat Abdominal Surgery Procedure

The entire animal-related processes were approved by the Institutional Animal Care and Use Committee of Chang Gung University (CGU107-220). Eighteen Wistar rats (weighing approximately 200–300 g) were enrolled for the study, and the animals were handled following the guidelines and regulations of the Ministry of Health and Welfare, Taiwan. Nine animals were employed for the in vivo pharmaceutical elution investigation, and nine rats were allocated for the activity survey.

**Figure 2:**
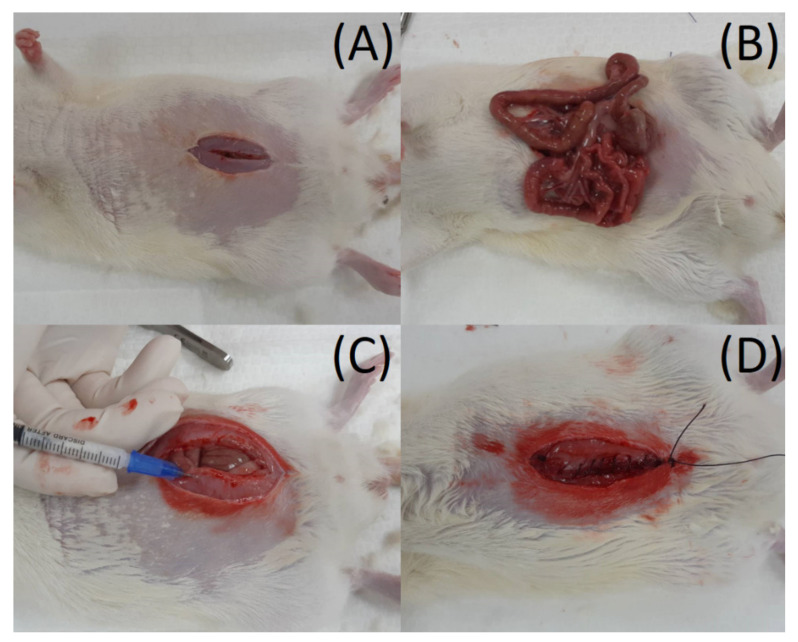
(**A**) A 5 cm-long skin incision wound was made at the middle of the abdominal wall. (**B**) The abdominal muscles were dissected until the exposure of the intra-abdominal organs, such as the stomach, intestine, liver, etc. (**C**) Drugs-loaded microparticles were implanted at the muscles of the abdominal wall. (**D**) The muscle, fascia, and skin were sutured with 3–0 Vicryl sutures.

The rats were first anesthetized via inhaling isoflurane within an anesthetic box. Anesthesia was maintained during the entire surgical procedure by mask inhalation with isoflurane. A 5 cm cut wound was achieved at the middle of the abdominal wall, and the abdominal muscle was dissected until the exposure of the intra-abdominal organs, such as the stomach, intestine, liver, etc. For in vivo pharmaceutical discharge experiments, the drug concentration near the tissue and in the drug serum levels was evaluated. To measure drug levels near the tissue, three rats were arranged to receive general anesthesia and abdominal surgery (*n* = 3). PLGA microparticles embedded with ropivacaine and dexamethasone were implanted into the muscle layer near the laparotomy wound. The muscle, fascia, and skin were then closed by 3–0 Vicryl sutures layer-by-layer (Figure 2). The rats were sent to the cage to observe their postoperative condition. Abdominal wall muscle tissue (approximately 50 mg) around the target sites was gathered on the 1st, 2nd, 7th, and 14th days after the operation. The drug level was evaluated by HPLC, and the wound was sealed by 3–0 Vicryl suture.

To assess drug concentrations in the blood, another six rats were injected with PLGA microparticles embedded with ropivacaine and dexamethasone into the abdominal muscle layer. Three rats (*n* = 3) each time were sacrificed 1 day post-operation and 3 days post-operation, and their blood was sampled for drug level analysis.

### 2.7. Post-Surgery Activity Assessment

**Figure 3:**
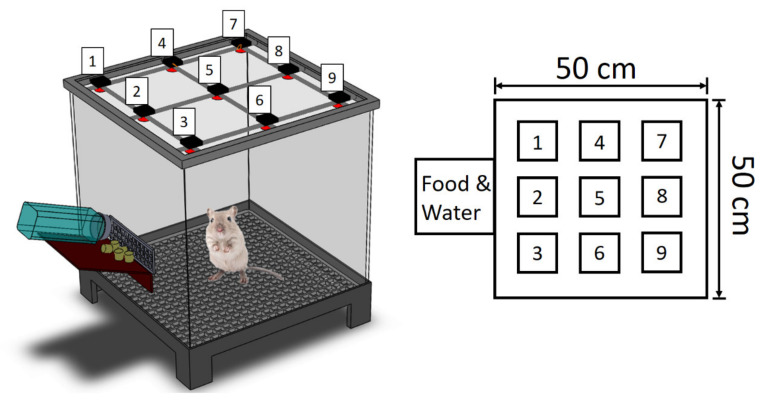
A schematic model of the cage used to evaluate the animal activity.

The nine rats allocated for the activity survey were separated into three groups (three rats in every group; *n* = 3). The rats of group A received no operation. This group served as a control group. Group B received abdominal surgery only, without drug administration. Group C received the abdominal surgery and the implantation with ropivacaine/dexamethasone loaded PLGA microparticles into the muscle layer near the wound. After the operations, the animal activity in distinct groups was assessed utilizing an activity cage (Figure 3) with a size of 50 cm × 50 cm × 50 cm. Nine photoelectric switch sensors (HP100-A1; Azbil Corp., Tokyo, Japan) were implemented above the cage. The sensors monitored the movements of each animal inside the cage. When the rat migrates from one zone of the cage to another, the sensor in the “approaching” zone is then prompted. A microprocessor that includes an accession interface was utilized to estimate the total prompting counts. The activity of each rat was surveyed for 7 days.

### 2.8. Statistics and Data Analysis

All experimental data were evaluated via one-way analysis of variance. The difference was considered statistically significant at *p*-values below 0.05.

## 3. Results

### 3.1. Assessment of Sprayed Microparticles

**Figure 4:**
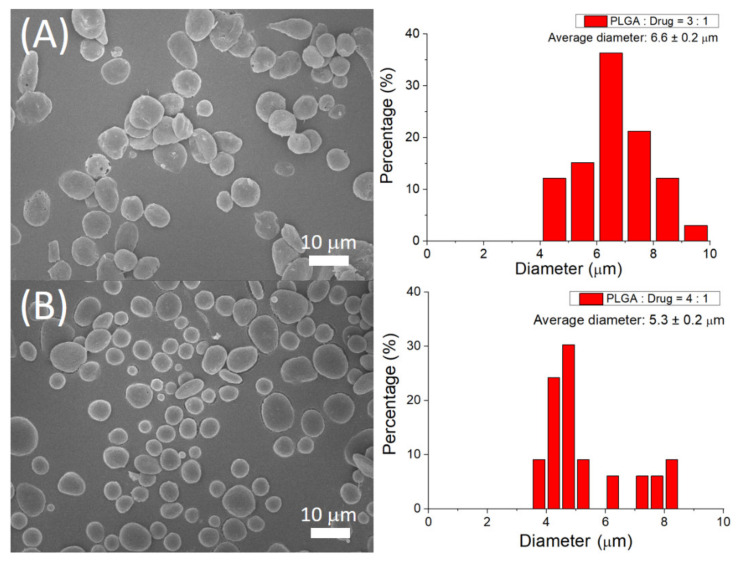
Scanning electron microscopy image and particle size distribution of electrosprayed microparticles with PLGA:drugs ratio of (**A**) 3:1 and (**B**) 4:1. Scale bar = 10 µm.

Figure 4 displays the SEM photos of sprayed microparticles. The calculated size distributions of microparticles with polymer-to-drug ratios of 3:1 and 4:1 were 6.6 ± 0.2 µm and 5.3 ± 0.2 µm, respectively.

**Figure 5:**
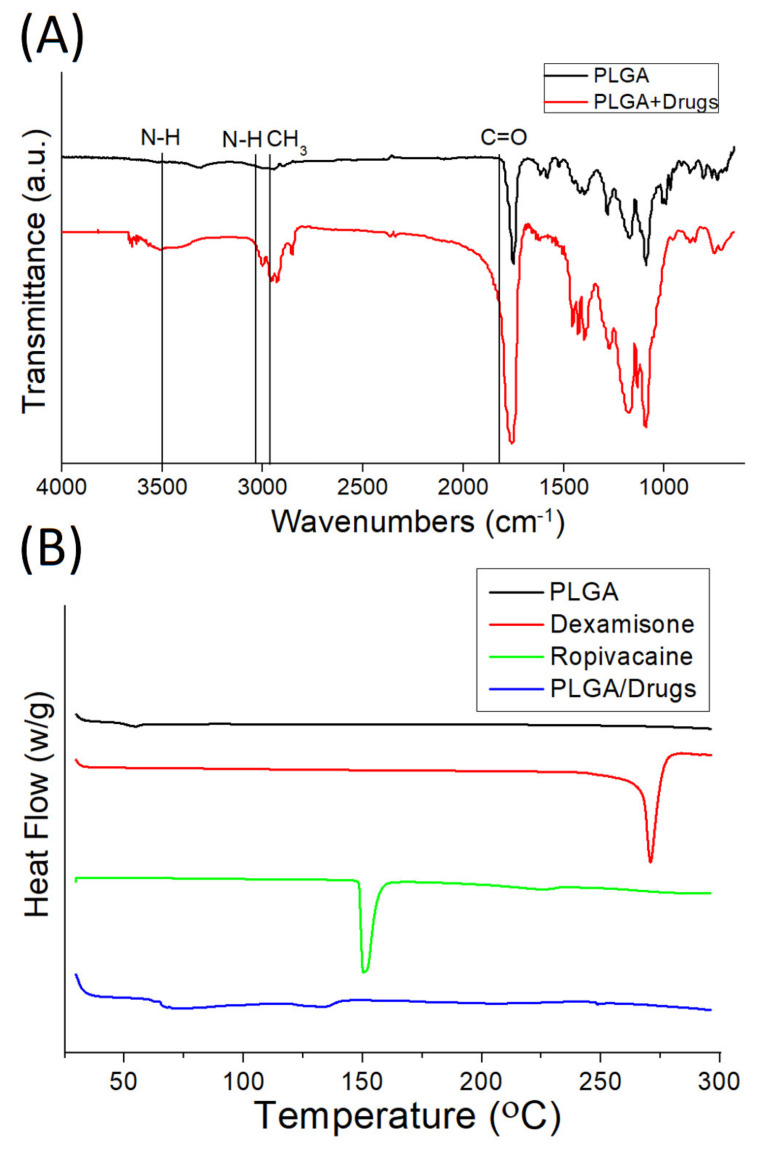
(**A**) Fourier transform infrared spectra. (**B**) Thermogram of pure PLGA and drug-loaded PLGA microparticles.

Figure 5a shows the obtained FTIR spectra of pure PLGA and drug-incorporated PLGA particles. The fresh peaks at 3000 cm^−1^ and 3490 cm^−1^ are attributed to the N-H vibrations of ropivacaine [21]. The enhanced absorption at 1760 cm^−1^ is correlated to C=O bonds, primarily owing to the presence of the pharmaceuticals [22]. Furthermore, the peak close to 2930 cm^−1^ may be a result of the CH_3_ bond intensification in incorporated drugs. Figure 5b displays the thermal properties of virgin PLGA and ropivacaine/dexamethasone-loaded PLGA microparticles. The peaks of ropivacaine and dexamethasone, at 151 °C and 270 °C, respectively, disappeared after being embedded into the PLGA matrix [23,24]. All these demonstrated the successful embedment of the pharmaceuticals in PLGA particles.

### 3.2. In Vitro Discharge of Analgesics-Loaded Particles

**Figure 6:**
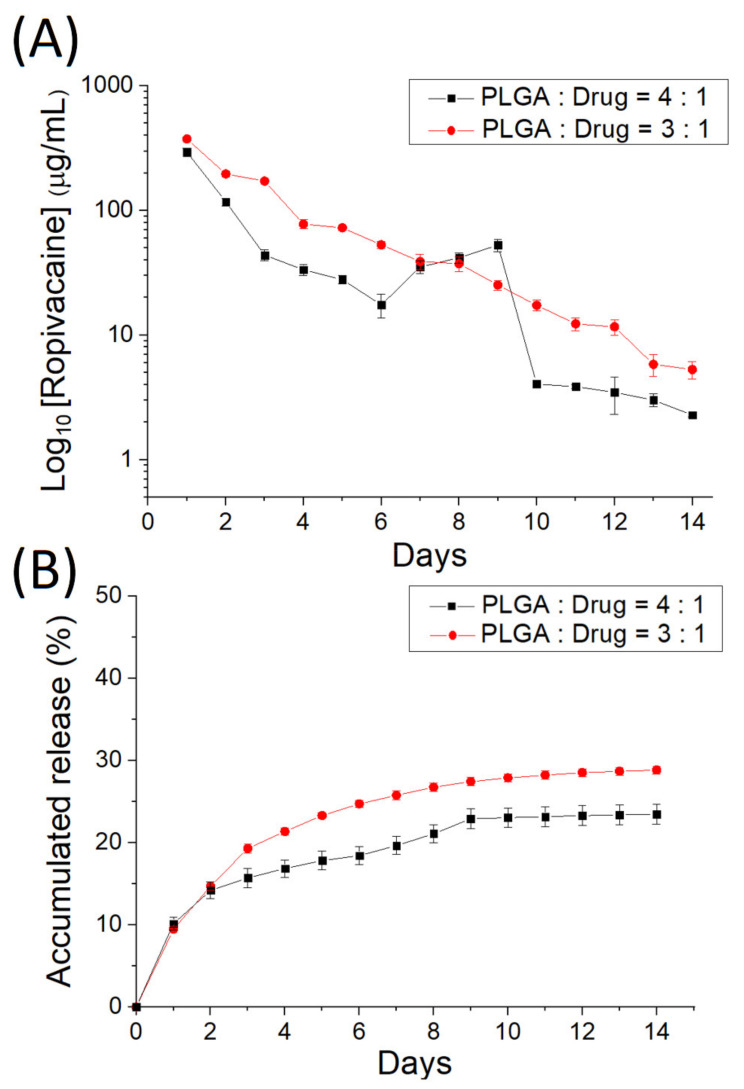
(**A**) Daily and (**B**) cumulative release curves of ropivacaine from the electrosprayed microparticles.

**Figure 7:**
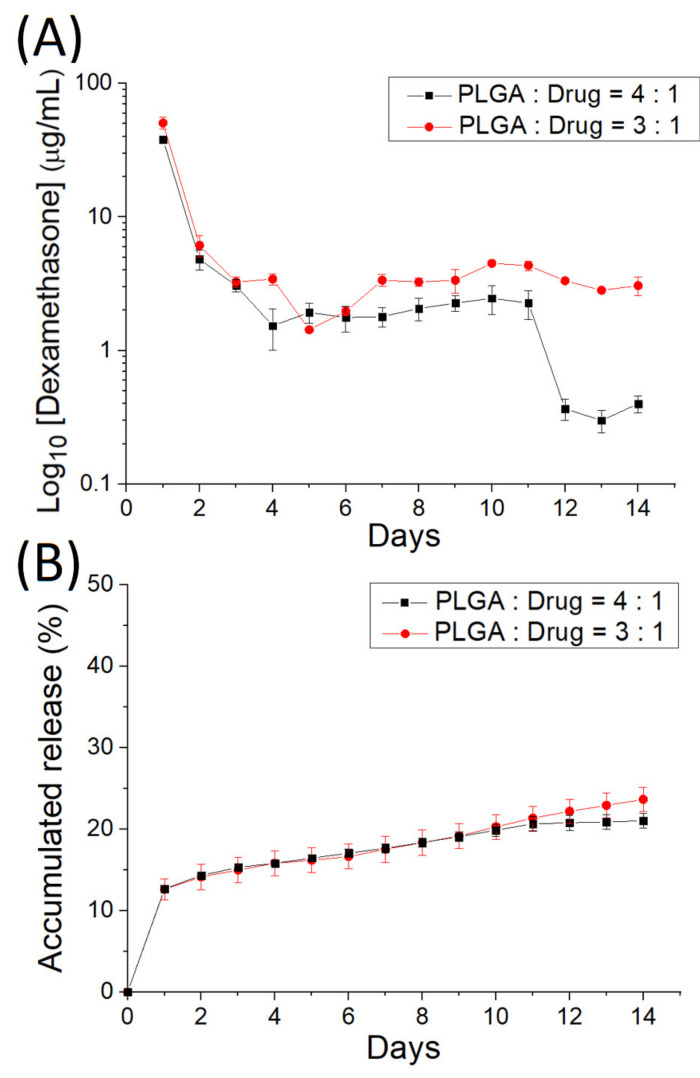
(**A**) Daily and (**B**) cumulative release curves of dexamethasone from the electrosprayed microparticles.

Figure 6 denotes the in vitro daily and cumulative elution characteristics of ropivacaine from the electrosprayed analgesic-loaded microparticles, and Figure 7 displays the release behavior of dexamethasone from the microparticles. Two-stage discharge patterns were noted with a burst on the first day and with a moderately diminishing release thereafter. PLGA particles discharged high levels of ropivacaine and dexamethasone [23,24] for over 14 days.

### 3.3. In Vivo Elution

**Figure 8:**
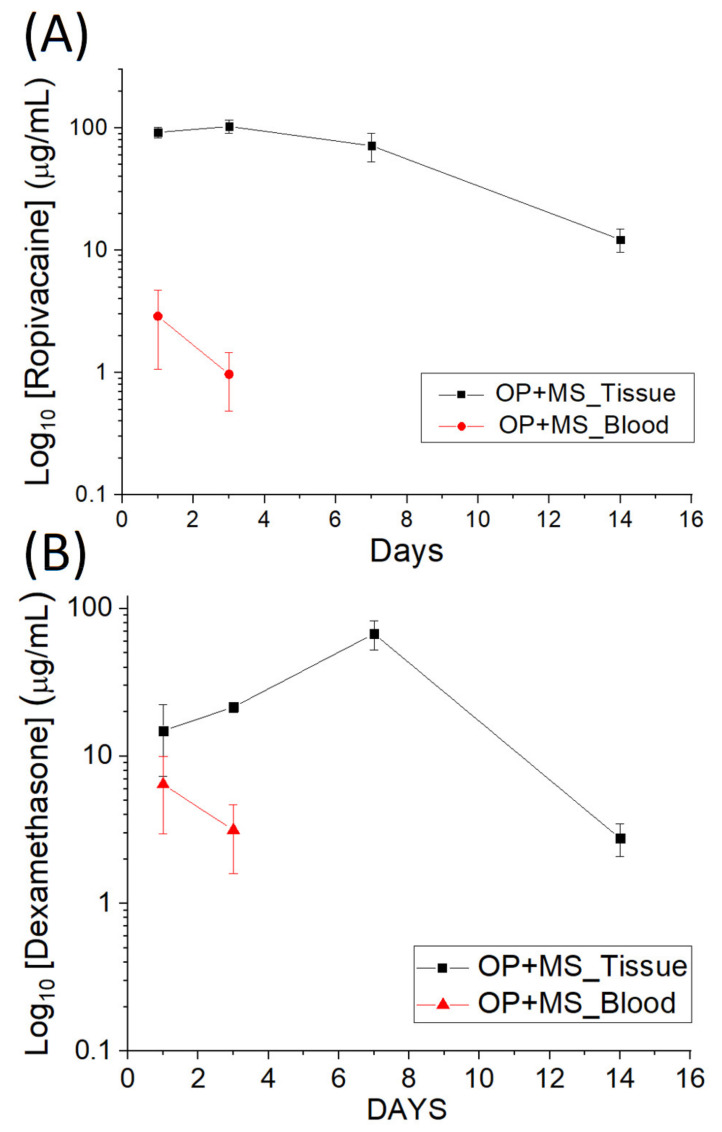
In vivo release curves of (**A**) ropivacaine and (**B**) dexamethasone.

Figure 8 displays the evaluated drug levels in tissue adjacent to the surgery site. Drug-loaded particles provided sustained elution of high concentrations of ropivacaine and dexamethasone in the tissues for over 14 days, and the drug concentrations in the blood of rats remained low.

### 3.4. Efficacy of Release Drugs in Pain Relief

**Figure 9:**
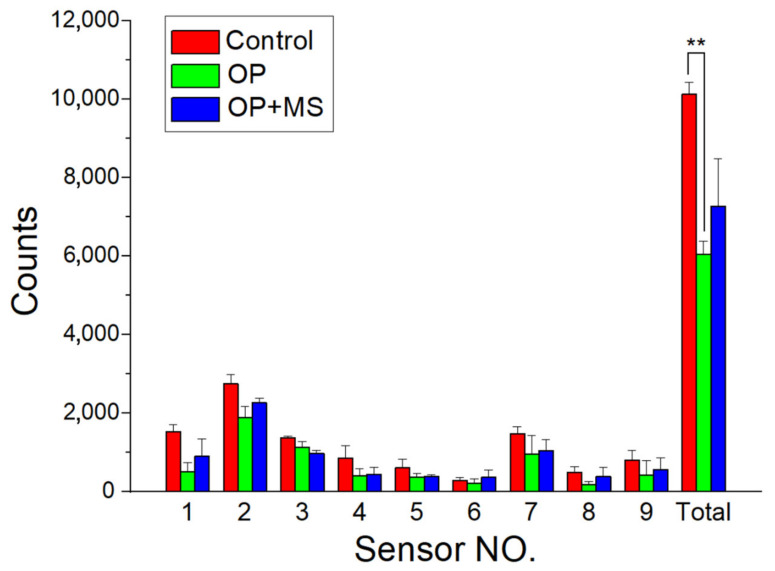
Monitored rat activity in the cage. Group A (red columns) was the control untreated group. Group B (green columns) received operation without any medication. Group C (blue columns) received surgery followed by the administration of drugs-loaded microparticles to the wound site (** *p* < 0.01).

The activity counts of monitored rats are illustrated in Figure 9. Sensor 1 has the greatest number of triggers, mainly owing to the animals’ persistent visits to the site of food and water supply. The total numbers of triggers were 10,119 ± 312, 6035 ± 339, and 7265 ± 1218 in the control group, the OP (surgery only) group, and the OP + MS (surgery + implantation of drug-loaded microparticles) group, respectively. The rat activity in the control group was significantly greater than that in the OP group (*p* < 0.01), underlining the unfavorable influence of the operation on the animals’ activity. The animals that had received the implantation of drug-loaded microparticles showed a higher level of activity than the OP group, demonstrating that the effectiveness of multimodal ropivacaine/dexamethasone treatment via microparticles in promoting animals’ activity levels post-operation.

## 4. Discussion

Distinct biomedical materials have been synthesized [25] to facilitate the therapeutic strategy of local drug delivery [26,27,28,29,30,31]. PLGA-based particulate systems are suitable for the controlled release of micromolecules and macromolecular biotherapeutics. These drug-loaded particulate systems (having particle diameters ranging from 1 to 1000 μm) can be injected or implanted adjacent to the targeted organs to maintain a relatively high drug concentration at the desired locations with a minimized drug distribution into other untargeted and healthy organs/tissues [25,32,33]. The degradable polymeric microparticles as a drug delivery strategy possess benefits over other systems because they do not need surgery for their application or removal from the body. Furthermore, the particles have shown excellent stability in the biological environment. The highly replicable production methods also offer support to incorporate hydrophilic and hydrophobic pharmaceuticals, granting them a wide variety of therapeutic applications [34]. When compared with conventional drug administrations, the local delivery of pharmaceuticals by the PLGA microparticles can prolong the delivery duration, increase the concentration at the target site, and minimize the systemic side effects of the drug [35,36,37].

Bhattacharjee et al. [38] proposed that, by adopting a particulate drug-delivery system, even though drug concentration in tissue raises rapidly, the serum concentration does not increase proportionally. However, the unpredictable and uncontrolled drug release remains a challenge, particularly from the perspective of developing sustained release formulations. The potential burst effect may also lead to overdose and toxicity of the drug. Bouriche et al. [39] showed an unexpected decrease in bioavailability of the drug from the microparticles compared with the oral dosage form of the same drug. Additionally, local anesthetics can not only reduce the pain but may also lead to some side effects, such as nerve injury or local anesthetic systemic toxicity [40]. Masters et al. [41] showed that polymers embedded with a local anesthetic lead to sensory and motor block for one week.

Unlike nerve block by local anesthetics used in limb surgery, nerve block used in abdominal surgery has less concern with motor block. However, abdominal surgery produces a huge midline laparotomy wound at the surface, leading to moderate to severe pain, which causes increased morbidity, hospitalization, and expenses [42]. An ideal protocol for postoperative pain management is highly desired.

Various analgesic protocols have been proposed for the management of huge laparotomy wounds, including epidural local anesthetics, peripheral nerve block by local anesthetics, and administration of analgesics such as opioids via intravenous or intramuscular injection [43,44,45]. Generally, epidural analgesia with local anesthetics has good efficacy for postoperative pain control. However, the procedure requires epidural catheter implantation and can lead to serious complications that are difficult to handle, such as dural puncture, infection, epidural hematoma, and central nerve injury. Intravenous or intramuscular injection of opioids also has good analgesic effectiveness but can easily cause opioid-related side effects, including nausea, vomiting, decreased bowel movement, etc. Peripheral nerve block, when combined with multimodal analgesia [46,47], has been proved to achieve good analgesic efficacy with reduced side effects.

In this study, we successfully developed ropivacaine- and dexamethasone-incorporated PLGA microspheres that offer extended drug discharge for postoperative pain control utilizing the electrospraying method [48]. PLGA is one of the most widely investigated biomaterials due to its ability to adapt to various activities, its adjustable biodegradability, and its superior biocompatibility. The material has also been vastly studied for target specificity and controlled release of pharmaceuticals and biomolecules [49].

In general, the release of pharmaceuticals from drug-loaded degradable microparticles consists of three different stages, namely a preliminary burst, a diffusion-controlled discharge, and a degradation-governed elution. After the electrospraying procedure, despite a majority of pharmaceuticals being embedded inside the matrix of PLGA microparticles, a few drug compounds may be allocated on the exterior of the particles, leading to a burst release. Thereafter, the pharmaceutical release patterns were governed simultaneously by diffusion and polymeric material degradation. Therefore, a steadily diminishing release of analgesics was noted. Overall, the PLGA particles were able to discharge high concentrations of ropivacaine and dexamethasone at the target location for over 14 days. They also offered consistent pain relief at the wound sites of studied animals without obvious motor dysfunction or systemic complications, offering benefits for extended postoperative pain control.

Microparticles have been demonstrated to promote therapeutic efficiency [25,26] due to the appropriate prevention of pharmaceuticals and molecules from degradation and the capability to implement the controlled discharge at the target zone. To tailor the transport and discharge of pharmaceuticals, it is essential to adjustably create the polymer particles with required sizes and size distributions. The small size deviation of electrosprayed microparticles demonstrated the excellent capability of the electrospraying process in preparing the drugs-loaded particles [3,4]. The ropivacaine and dexamethasone-incorporated particles exploited in this work, owing to their tiny dimension (approximately 5 µm), can be amiably delivered into the body by injection via a syringe. Therefore, the drug-loaded particles can be adopted for various pain-management strategies, providing the benefit of good pain control with improved life quality. Furthermore, the empirical outcomes showed a significant enhancement of total triggers (*p* < 0.01) at one week after the operation for the rats that received the implantation of ropivacaine- and dexamethasone-incorporated particles relative to those without implantation. This further demonstrates the effectiveness of ropivacaine and dexamethasone-incorporated microparticles in post-surgery pain management.

Despite the preliminary findings, there are limitations in this study. First, the number of animals included in this work was relatively low. Another limitation is the restricted period of activity survey. Thirdly, the correlation between the discoveries in animals here and the pain management in human beings is unknown and needs additional investigations. These may require some further explorations.

## 5. Conclusions

This study has successfully produced degradable, drug-eluting PLGA microparticles via the electrospraying technique to offer extended discharge of ropivacaine and dexamethasone to the target site. Sprayed particles could deliver high concentrations of ropivacaine and dexamethasone for more than 14 days. Rats implanted with drugs-embedded particles demonstrated superior activity compared with the rats without implantation. Electrosprayed drug-eluting particles showed their efficacy and long-term pain relief for the postoperative healing process. Eventually, the drugs loaded-microparticles can be employed for post-surgery pain management in humans.

## Data Availability

The data presented in this study are available on request from the corresponding author.

## References

[1] Bale S., Khurana A., Reddy A.S.S., Singh M., Godugu C. (2016). Overview on therapeutic applications of microparticulate drug delivery systems. Crit. Rev. Ther. Drug Carr. Syst..

[2] Lengyel M., Kállai-Szabó N., Antal V., Laki A.J., Antal I. (2019). Microparticles, microspheres, and microcapsules for advanced drug delivery. Sci. Pharm..

[3] Gaskell S.M. (1997). Electrospray: Principles and practice. J. Mass Spectrom..

[4] Jaworek A. (2007). Micro- and nanoparticle production by electrospraying. Powder Technol..

[5] Wang J., Jansen J.A., Yang F. (2019). Electrospraying: Possibilities and challenges of engineering carriers for biomedical applications—A mini review. Front. Chem..

[6] Hsu M.Y., Feng C.H., Liu Y.W., Liu S.J. (2019). An orthogonal model to study the effect of electrospraying parameters on the morphology of poly (d, l)-lactide-co-glycolide (PLGA) particles. Appl. Sci..

[7] Lee D., Hsu M.Y., Li M.J., Liu Y.W., Liu S.J., Liu C.Y., Ito H. (2018). Factors affecting the co-axial electrospraying of core–shell-structured poly(d, l-lactide-co-glycolide) microparticles. Japan J. Appl. Phys..

[8] Geeraerts T., Velly L., Abdennour L., Asehnoune K., Audibert G., Bouzat P., Bruder N., Carrillon R., Cottenceau V., Cotton F. (2018). Management of severe traumatic brain injury (first 24 hours). Anaesth. Crit. Care Pain Med..

[9] Chou R., Gordon D.B., de Leon-Casasola O.A., Rosenberg J.M., Bickler S., Brennan T., Carter T., Cassidy C.L., Chittenden E.H., Degenhardt E. (2016). Management of Postoperative Pain: A Clinical Practice Guideline From the American Pain Society, the American Society of Regional Anesthesia and Pain Medicine, and the American Society of Anesthesiologists’ Committee on Regional Anesthesia, Executive Committee, and Administrative Council. J. Pain.

[10] Tano P.F., Apiribu F., Tano E.K., Boamah Mensah A.B., Dzomeku V.M., Boateng I. (2021). Predicting factors that determine patients’ satisfaction with post-operative pain management following abdominal surgeries at Komfo Anokye Teaching Hospital, Kumasi, Ghana. PLoS ONE.

[11] Kjølhede P., Bergdahl O., Wodlin N.B., Nilsson L. (2019). Effect of intrathecal morphine and epidural analgesia on postoperative recovery after abdominal surgery for gynecologic malignancy: An open-label randomised trial. BMJ. Open.

[12] Ganapathy S. (2012). Wound/intra-articular infiltration or peripheral nerve blocks for orthopedic joint surgery: Efficacy and safety issues. Curr. Opin. Anaesthesiol..

[13] Kau Y.-C., Liao C.-C., Chen Y.-C., Liu S.-J. (2014). Sustained release of lidocaine from solvent-free biodegradable poly[(d, l)-lactide-co-glycolide] (PLGA): In vitro and in vivo study. Materials.

[14] Durst M.S., Arras M., Palme R., Talbot S.R., Jirkof P. (2021). Lidocaine and bupivacaine as part of multimodal pain management in a C57BL/6J laparotomy mouse model. Sci. Rep..

[15] Miller R.D., Pardo M., Stoelting R.K. (2011). Basics of Anesthesia.

[16] Cummings K.C., Napierkowski D.E., Parra-Sanchez I., Kurz A., Dalton J.E., Brems J.J., Sessler D.I. (2011). Effect of dexamethasone on the duration of interscalene nerve blocks with ropivacaine or bupivacaine. Br. J. Anaesth..

[17] Huynh T.M., Marret E., Bonnet F. (2015). Combination of dexamethasone and local anaesthetic solution in peripheral nerve blocks: A meta-analysis of randomised controlled trials. Eur. J. Anaesthesiol..

[18] Mao Y., Zuo Y.M., Mei B., Chen L.J., Liu X.S., Zhang Z., Gu E.W. (2018). Efficacy of perineural dexamethasone with ropivacaine in thoracic paravertebral block for postoperative analgesia in elective thoracotomy: A randomized, double-blind, placebo-controlled trial. J. Pain Res..

[19] Peng Y.J., Kau Y.C., Wen C.W., Liu K.S., Liu S.J. (2010). Solvent-free biodegradable scleral plugs providing sustained release of vancomycin, amikacin and dexamethasone–An in vivo study. J. Biomed. Mater. Res. A.

[20] Li A., Yang F., Xin J., Bai X. (2019). An efficient and long-acting local anesthetic: Ropivacaine-loaded lipid-polymer hybrid nanoparticles for the control of pain. Int. J. Nanomed..

[21] Martins M.L., Eckert J., Jacobsen H., dos Santos E.C., Ignazzi R., de Araujo D.R., Bellissent-Funel M.-C., Natali F., Koza M.M., Matic A. (2017). Raman and Infrared spectroscopies and X-ray diffraction data on bupivacaine and ropivacaine complexed with 2-hydroxypropyl−β−cyclodextrin. Data Brief.

[22] Chiang Z.-C., Yu S.-H., Chao A.-C., Dong G.-C. (2012). Preparation and characterization of dexamethasone-immobilized chitosan scaffold. J. BioSci. Bioeng..

[23] Rodrigues L.B., Leite H.F., Yoshida M.I., Saliba J.B., Cunha A.S., Faraco A.A.G. (2009). In vitro release and characterization of chitosan films as dexamethasone carrier. Int. J. Pharm..

[24] Zhai Y., Xu R., Wang Y., Liu J., Wang Z., Zhai G. (2014). Ethosomes for skin delivery of ropivacaine: Preparation, characterization and ex vivo penetration properties. J. Liposome Res..

[25] Alavi M., Webster T.J. (2021). Recent progress and challenges for polymeric microsphere compared to nanosphere drug release systems: Is there a real difference?. Bioorg. Med. Chem..

[26] Verma D., Bhatia A., Chopra S., Dua K., Prasher P., Gupta G., Tambuwala M.M., Chellappan D.K., Aljabali A.A.A., Sharma M. (2021). Advancements on microparticles-based drug delivery systems for cancer therapy. Advanced Drug Delivery Systems in the Management of Cancer.

[27] Jusu S.M., Obayemi J.D., Salifu A.A., Nwazojie C.C., Uzonwanne V., Odusanya O.S., Soboyejo W.O. (2020). Drug-encapsulated blend of PLGA-PEG microspheres: In vitro and in vivo study of the effects of localized/targeted drug delivery on the treatment of triple-negative breast cancer. Sci. Rep..

[28] Salapa J., Bushman A., Lowe K., Irudayaraj J. (2020). Nano drug delivery systems in upper gastrointestinal cancer therapy. Nano Converg..

[29] Brigham N.C., Ji R.-R., Becker M.L. (2021). Degradable polymeric vehicles for postoperative pain management. Nat. Commun..

[30] Bajwa S.J.S. (2021). Dexmedetomidine and ketamine - Comrades on an eternal journey!. Indian J. Anaesth..

[31] Sabuj M.Z.R., Islam N. (2021). Inhaled antibiotic-loaded polymeric nanoparticles for the management of lower respiratory tract infections. Nanoscale Adv..

[32] Langer R. (1998). Drug delivery and targeting. Nature.

[33] Allen T.M., Cullis P.R. (2004). Drug Delivery Systems: Entering the Mainstream. Science.

[34] Davoodi P., Lee L.Y., Xu Q., Sunil V., Sun Y., Soh S., Wang C.-H. (2018). Drug delivery systems for programmed and on-demand release. Adv. Drug Deliv. Rev..

[35] Patil N.V., Wadd N.V., Thorat S.S., Upadhye S.S. (2020). Microspheres: A novel drug delivery system. Am. J. PharmTech Res..

[36] Jain N.K. (2011). Controlled and Novel Drug Delivery.

[37] Alagusundaram M., Madhu S.C., Umashankari K., Attuluri V.B., Lavanya C., Ramkanth S. (2009). Microspheres as a novel drug delivery system-A review. Int. J. ChemTech Res..

[38] Bhattacharjee S. (2021). Understanding the burst release phenomenon: Toward designing effective nanoparticulate drug-delivery systems. Ther. Deliv..

[39] Bouriche S., Alonso-García A., Cárceles-Rodríguez C.M., Rezgui F., Fernández-Varón E. (2021). An in vivo pharmacokinetic study of metformin microparticles as an oral sustained release formulation in rabbits. BMC Veter-Res..

[40] Thiele E.L., Nemergut E.C. (2020). Miller’s Anesthesia.

[41] Masters D.B., Berde C.B., Dutta S.K., Griggs C.T., Hu D., Kupsky W., Langer R. (1993). Prolonged Regional Nerve Blockade by Controlled Release of Local Anesthetic from a Biodegradable Polymer Matrix. Anesthesiology.

[42] Gan T.J. (2017). Poorly controlled postoperative pain: Prevalence, consequences, and prevention. J. Pain Res..

[43] Chen Q., Chen E., Qian X. (2021). A narrative review on perioperative pain management strategies in enhanced recovery pathways—The past, present and future. J. Clin. Med..

[44] Gathege D., Abdulkarim A., Odaba D., Mugambi S. (2021). Effectiveness of pain control of local anaesthetic wound infusion following elective midline laparotomy: A randomized trial. World J. Surg..

[45] Cindea I., Balcan A., Gherghina V., Nicolae G., Samoila B., Costea D. (2011). Postoperative pain management after major abdominal surgery in elderly patients. Eur. J. Anaesthesiol..

[46] Gelman D., Gelmanas A., Urbanaitė D., Tamošiūnas R., Sadauskas S., Bilskienė D., Naudžiūnas A., Širvinskas E., Benetis R., Macas A. (2018). Role of multimodal analgesia in the evolving enhanced recovery after surgery pathways. Medicina.

[47] Shen L., Huang Y.-G. (2016). Role of postoperative multimodal analgesia in abdominal and pelvic enhanced recovery after surgery. Acad. Med. Sin..

[48] Ramakrishna S., Zamani M., Prabhakaran M.P. (2013). Advances in drug delivery via electrospun and electrosprayed nanomaterials. Int. J. Nanomed..

[49] Makadia H.K., Siegel S.J. (2011). Poly lactic-co-glycolic acid (PLGA) As biodegradable controlled drug delivery carrier. Polymers.

